# Efficacy of intra-articular hyaluronic acid injections in hip osteoarthritis: a meta-analysis of randomized controlled trials

**DOI:** 10.18632/oncotarget.20995

**Published:** 2017-09-18

**Authors:** Bei Wu, Yao-Min Li, Yan-Cheng Liu

**Affiliations:** ^1^ Department of Nuclear Medicine, Tianjin Hospital, Tianjin, P.R. China; ^2^ Department of Rehabilitation, Tianjin Hospital, Tianjin, P.R. China; ^3^ Department of Spine Surgery, Tianjin Hospital, Tianjin, P.R. China

**Keywords:** hyaluronic acid, meta-analysis, osteoarthritis, hip

## Abstract

There is less credible evidence of using of intra-articular injections of hyaluronic acid (HA) to treat hip osteoarthritis (OA). This study is to determine the therapeutic effects and risk of adverse events of HA administration for hip OA. The MEDLINE, Cochrane of Systematic Reviews, Cochrane Clinical Trial Register and EMBASE, were searched for articles published. Eligible studies were limited to trials of HA with a randomized design. A total of six studies were included in this the meta-analysis. The pooled effect size of improved pain scores from pretreatment was –0.72 (95%CI; –1.06 to –0.39; *P* < 0.05). The standardized mean difference (SMD) of improved Lequesne's index and McMaster Universities Osteoarthritis Index (WOMAC) was –0.74 (95%CI, –1.42 to –0.51; *P* < 0.05) and –7.75 (95%CI, –14.28 to –1.21; *P* < 0.05), respectively. The pooled effect size of improved pain scores compared HA with different controls was 0.03 (95%CI; –0.20 to 0.26; *P* < 0.05). The SMD of improved Lequesne's index and WOMAC was –0.24 (95%CI, –0.50 to 0.02; *P* > 0.05) and –0.13 (95%CI, 0.64 to 0.37; *P* > 0.05). There were no significant differences between HA and control group in adverse events (RR: 0.94; 95%CI, 0.41 to 2.20; *P* > 0.05). Intra-articular HA in hip OA can significantly reduce pain and improve functional recovery when compared with the condition before treatment. However, there seems no significant difference between HA and saline or other treatments. Currently, available evidence indicated that intra-articular HA in hip OA would not be increased risk of adverse events.

## INTRODUCTION

Osteoarthritis (OA) is a disorder which characterized by focal areas of damage to articular cartilage at weight-bearing areas, causing pain and disability, most common in the elderly. Osteoarthritis associated with changes in the subchondral bone, formatted of the cyst, synovitis, osteophyte formation, joint space losing, due to cartilage loss and joint capsule thickening [1, 2]. The prevalence ranges of hip OA is from 3% to 11% in populations older than 35 years, which is the second place occurrence of OA affecting a large joint [3]. Moreover, the socioeconomic costs on the hip OA have been increased by 80% in the past ten years [1, 4].

Current therapies for hip OA include a combination of nonpharmacologic and pharmacologic treatments [5, 6]. Surgery is considered as a last resort management option, appropriate for patients who are failed to benefit from other more conservative treatment options.. In spite of this, a plenty of possible problems including infection, blood clots, loosening, dislocation, nerve and blood vessel injury, and, not least of all, total hip replacement (THR) can be complicated by a high risk of mortality in elderly [7]. For pharmacologic treatments, the drug therapy is mainly symptomatic and includes simple analgesics, nonsteroidal anti-inflammatory drugs, intra-articular injection of glucocorticoids are standard clinical procedures at the first-line. But none of the methods have shown to detain pathology progression or reverse cartilage damage in patients [8].

Intra-articular injection of hyaluronic acid (HA) is a slow-acting drug for the treatment of symptoms of OA, which involves using the medical device. This treatment has recently become one of the favorite non-operative options for treating osteoarthritis and approved by the U.S. Food and Drug Administration in 1999 [9]. HA is a critical constituent of the healthy synovial fluid, which increases the viscosity of the synovial fluid. And HA has a significant contributor to the homeostasis of joints, which facilitate gliding via layer formation on the cartilage, and also soothes the pain and exerts an immunomodulatory effect on inflammatory cells. HA is acting as a shock absorbent to protect soft tissue from trauma. Besides, the function of protective effects on cartilage extracellular matrix have been reported by *in vitro* and *in vivo* studies, which could reduce the production and activity of proinflammatory mediators and matrix metallo proteinases [10, 11].

A numerous of knee OA studies published in the recently, which has been widely applied in the management with HA, and the effectiveness of treatment has been confirmed by several meta-analyses. The outcome of intra-articular HA treatment in hip joints is less documented because of some limitations on studies of hip OA. Including difficulty of administration, broad localization of the hip joint, indistinct descriptions for the injection site, proximity to the neurovascular structures and inexperience of the doctors on the technique [9, 12–14].

Therefore, the effectiveness of HA injection treatment for hip OA warrants a thorough investigation. There is even less credible evidence supporting intra-articular injection HA for treating hip OA. The objectives of this meta-analysis were to evaluate by the published randomized controlled trials (RCT) investigating the effectiveness of intra-articular HA injection for the treatment of hip OA and to establish whether it is an efficient and safety modality to prevent pain and improve joint function.

## MATERIALS AND METHODS

### Search strategy

**Figure 1:**
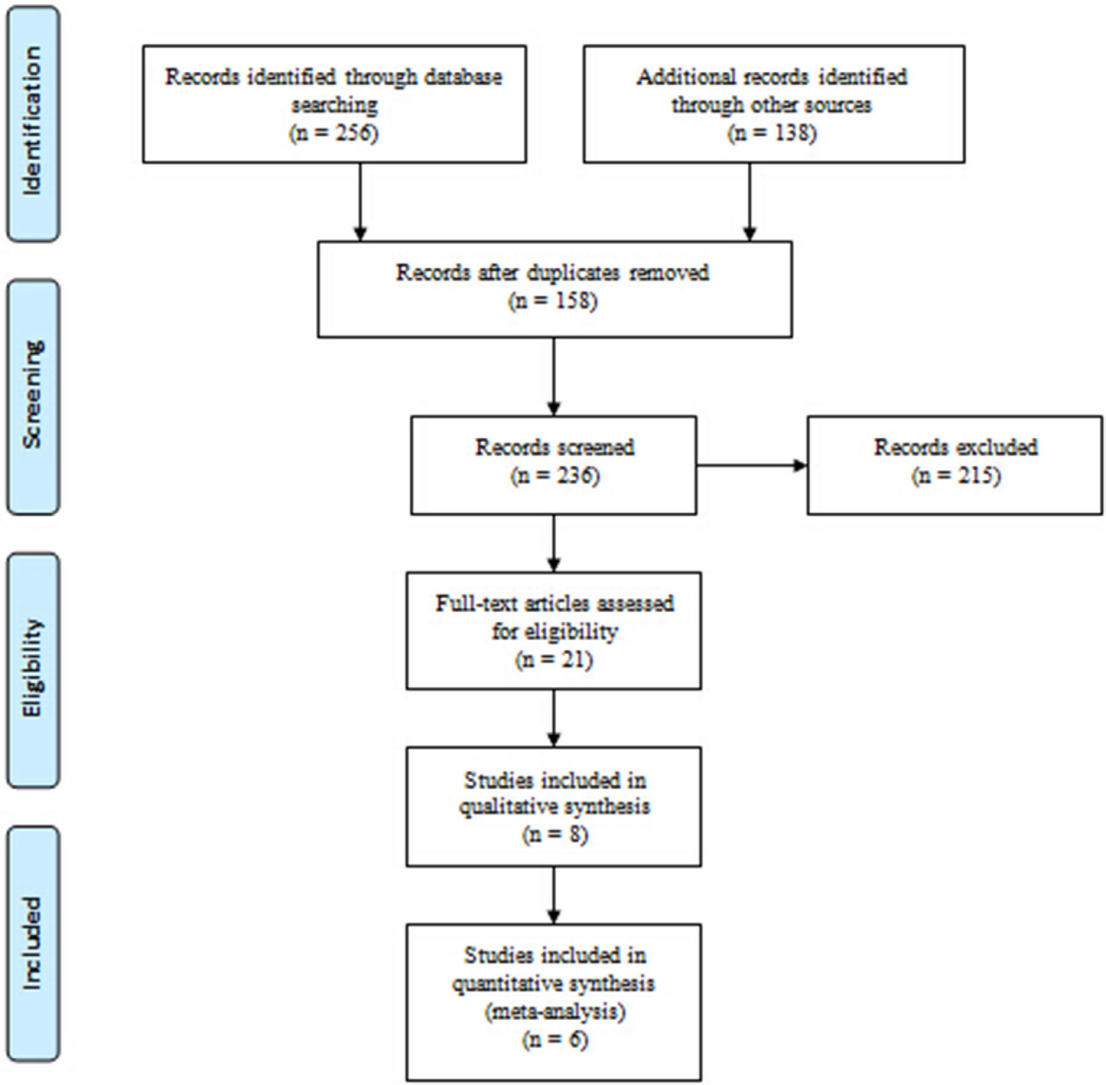
Flowchart of the study selection process

To assemble all of the relevant literature, we conducted a PRISMA (Preferred Reporting Items for Systematic Reviews and Meta- Analyses)-compliant search of MEDLINE database, Cochrane CENTRAL database, ScienceDirect, EMBASE and Google scholar for the relevant published studies to May 2017. To maximize the search specificity and sensitivity, we used following search terms: osteoarthritis, hip, hyaluronic acid, and viscosupplementation. Figure.1 presents search strategy, which only included studies conducted on humans for all published, unpublished and ongoing trials attempted to gather information on. In addition, the WHO International Clinical Trials Registry Platform, UK National Research Register Archive and Current Controlled Trials are used for a further search manually for articles.

### Selection criteria and quality assessment

The aim of this study was to evaluate the therapeutic effects of hyaluronic acid on osteoarthritis of the hip. The following included criteria: (1) prospective randomized phase II and III trials of patients with osteoarthritis of the hip, (2) studies that included the random assignment of participants to treatment with intra-articular HA or a control (mepivacaine, saline or placebo), (3) hip OA confirmed by clinical and radiologic assessment, and (4) reported outcomes including: numerical rating scale (NRS), visual analogue scale (VAS) pain, Lequesne index and Western Ontario and McMaster Universities Osteoarthritis Index (WOMAC) (5) more than 15 patients in the study or subgroup of interest. We also excluded studies that investigated the effectiveness of intra-articular HA for the treatment of hip osteochondral lesions. The Cochrane Collaboration’s tool was used for assessing the risk of bias with methodological quality of each RCTs.

### Study selection

Two independent authors performed the selection based on the eligibility criteria; full text would be read when the studies met the inclusion criteria. When the citation could not be excluded immediately, disagreements The third investigator resolved disagreements by consensus.

### Data extraction

Data extraction was conducted according to the PRISM statement and extracted eligible peer-reviewed articles by two authors. Data extraction discrepancies between the two reviewers were resolved by discussion and consensus or, if necessary, by third-party adjudication. Authors of the studies would be contacted for missing data or further information when it’s necessary. The following outcomes were extracted from the included publications: (1) demographic data of participants; (2) pain scale; (3) WOMAC; (4) Lequesne index; (5) adverse events mentioned in individual studies were involved.

### Assessment of methodological quality

Eligible articles were assessed for methodological quality by using the Cochrane Handbook for Systematic Reviews of Interventions 5.0 independently. The disagreements were resolved by discussion. When could not achieve the consensus, the third author was the adjudicator. The Cochrane collaboration’s tool for following fields: (1) blinding of outcome assessment; (2) allocation concealment; (3) blinding of participants and personnel; (4) details of randomization method; (5) incomplete outcome data; (6) selective outcome reporting and (7) other sources of bias, to provide a qualification of risk of bias.

### Statistical analysis

Extracted data were pooled for Stata 11.0 (for Windows (StataCorp LP, College Station, TX). For continuous outcomes, to evaluate the effectiveness of HA treatment compared with the condition before treatment, we used the standardized mean difference between the baseline and status after therapy as our measure of effectiveness. If necessary, data were extracted from the ratio of the difference between baseline and posttreatment pain to the SD of pooled results which did not directly refer to studies Because the pooled SD was calculated reality-on the rule of intention-to-treat so that the dropout rate was not considered. Therefore, the participant numbers between the baseline and post treatment kept constant from data sets [9, 14, 15].

We also established by detailed correlation between intra-articular HA injections and other controlled treatments. In such cases, the standardized mean difference of the change in pain between the HA and controlled groups was employed, and the corresponding effect size indicated the ratio of the difference of the changes in different outcomes between the HA and controlled groups to the pooled SD [9, 14, 15]. For dichotomous outcomes, the risk ratio (RR) or the odds ratio (OR) and 95% CI were assessed. A probability of *p* < 0.05 was considered to be statistically significant. The Definition of short term is occurring within three months or less and long term for occurring after six months. We calculated the pooled effect size at each time point respectively using a random effects model with between study variance analysis by above-mentioned method and An I^2^ statistic value of 50% was considered suggestive substantial heterogeneity. Fewer than ten studies did not assess publication bias by using a funnel plot diagram [16].

## RESULTS

### Search results

A total of 394 relevant studies evaluating osteoarthritis of the hip were preliminarily reviewed, of which 6 studies [17–22] eventually screened for the eligibility criteria and carefully selected for analysis reporting patients at final follow-up were eligible for data extraction and meta-analysis (Figure 1).

### Demographic characteristics

The demographic characteristics of the studies included are showed in Table 1. A total of six studies were included in this meta-analysis, the average age of participants ranged from 59.5 to 70 years (median of 65 years, reported in 5 studies). All 6 studies reported on Kellgren-Lawrence grades of radiographic severity: grade 1–2 was found in 27.4% of participants, and grade 3–4 in 72.6% of participants. The average length of follow-up ranged from 52 days to 180 days, and the average completeness of follow-up ranged from 81% to 94%. One study [20] compared injections of Mepivacaine with injection of HA, another study [17] compared the intra-articular HA administration to injection of G-F20. Two studies [18, 19] compared injections of HA with depomedrona and three studies [18, 19, 21] compared injections of HA with phosphate buffered saline control.

**Table 1:** Characteristcs of included studies

Study (years)	Country	Average age (years) (HA/Con)	Numerber of patients (HA/Con)	MW (kDa)	Product	Treatment strategy	Comparison	Guidance	Follow-up	Outcomes
Andrew (2010)	United States	59/59	156/156	Unclear	hylan G-F 20	2 ml, 2 weeks apart injection	methylprednisolone acetate	Fluoroscopy	6 months	5
Alberto (2009)	Italy	67.0/70.0	17/17	1500–3200	Hyalubrix	2 ml (15 mg), 2 monthly injection	Mepivacaine	Fluoroscopy	6 months	1,2,3,4,7
Canan (2005)	Turkey	58.8/60.4	25/18	1200–1400	Ostenil	2.0 ml, 1 weekly injection	Synvisc	Fluoroscopy	6 months	1,2,5,7
Ismaёl (2010)	Unite Kingdom	69.0/68.5	18/37	800	Unclear	3 ml (60 mg), A single injection	Depomedronaand NS	Fluoroscopy	52 days	5,6,7
Pascal (2008)	France	60.8/59.5	42/43	900	Adant	2.5 ml, a single injection	NS	Fluoroscopy	3 months	1,3,5,7
Qvistgaard (2006)	Denmark	65.0/66.5	33/68	1500–3200	Hyalubrix	2 ml, 3 ml injections in 2 weeks.	Depomedrol And NS	Fluoroscopy	3 months	1,3,5,7

HA, Hyaluronic acid group; Con, Control group; NS, normal saline.

Outcomes: 1. Visual analogue scale (VAS) pain 2.Lequesne’s index 3. Global assessment index 4.Nonsteroidal anti-inflammatory drug intake. 5. (Western Ontario and McMaster Universities Osteoarthritis Index) WOMAC 6. Numerical rating scale (NRS) 7. adverse events.

### Quality assessment

The results of the quality assessment are presented in Table 2. Three studies [19–21] stated the exact randomization methods and allocation concealment used. Only 3 studies [19–21] mentioned that appliance of blind method was employed both in the patients and the assessors. In addition, 3 studies [18, 20, 21] blinded only the patients, 1 studies [20] documented a high risk in incomplete outcome data.

**Table 2:** The methodological quality of the RCTs

Study (years)	Random sequence generation	Allocation concealment	Blinding of patients and personnel	Blinding of outcomes assessment	Incomplete outcome data	Selective reporting	Other bias
Andrew (2011)	Low risk	Low risk	Low risk	Low risk	Low risk	Low risk	Unclear
Alberto (2009)	Low risk	Low risk	Low risk	Low risk	Low risk	Low risk	Unclear
Canan (2005)	Unclear	Unclear	Unclear	Low risk	Low risk	Low risk	Unclear
Ismaёl (2010)	Low risk	Low risk	Unclear	Low risk	Low risk	Low risk	Unclear
Pascal (2008)	Low risk	Low risk	Low risk	Low risk	Low risk	Low risk	Unclear
Qvistgaard (2006)	Unclear	Unclear	Low risk	Low risk	Low risk	Low risk	Unclear

### Intra-articular injection of HA efficacy vs. pre-treatment

#### Pain scores

**Figure 2:**
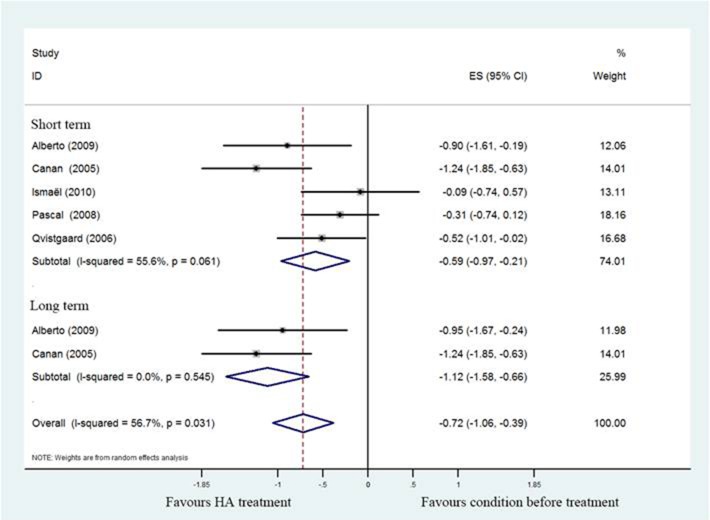
Forest plot showing different of effect size for pain between pretreatment and post-treatment

All studies reported pain related index for the HA group relative to the baseline at 1–6months, four studies [17, 19–21] used 100mm VAS scale and remained one study [18] used NRS for evaluating pain strength. The pooled effect size of pain scores was –0.72 (95%CI; –1.06 to –0.39; *P* < 0.05), with significant heterogeneity (I^2^ = 56.7%), and the subgroup analysis demonstrated that pooled effect size of hip pain in short-term was –0.52 (95%CI; –0.97 to –0.21), with significant heterogeneity (I^2^ = 55.6%) , and it was –1.12 (95%CI; –1.58 to –0.66) with no unexplained heterogeneity (I^2^ = 0%) in a long-term follow-up. That means patients who underwent HA injection had better pain scores than baseline status. (Figure 2).

#### Lequesne’s index

**Figure 3:**
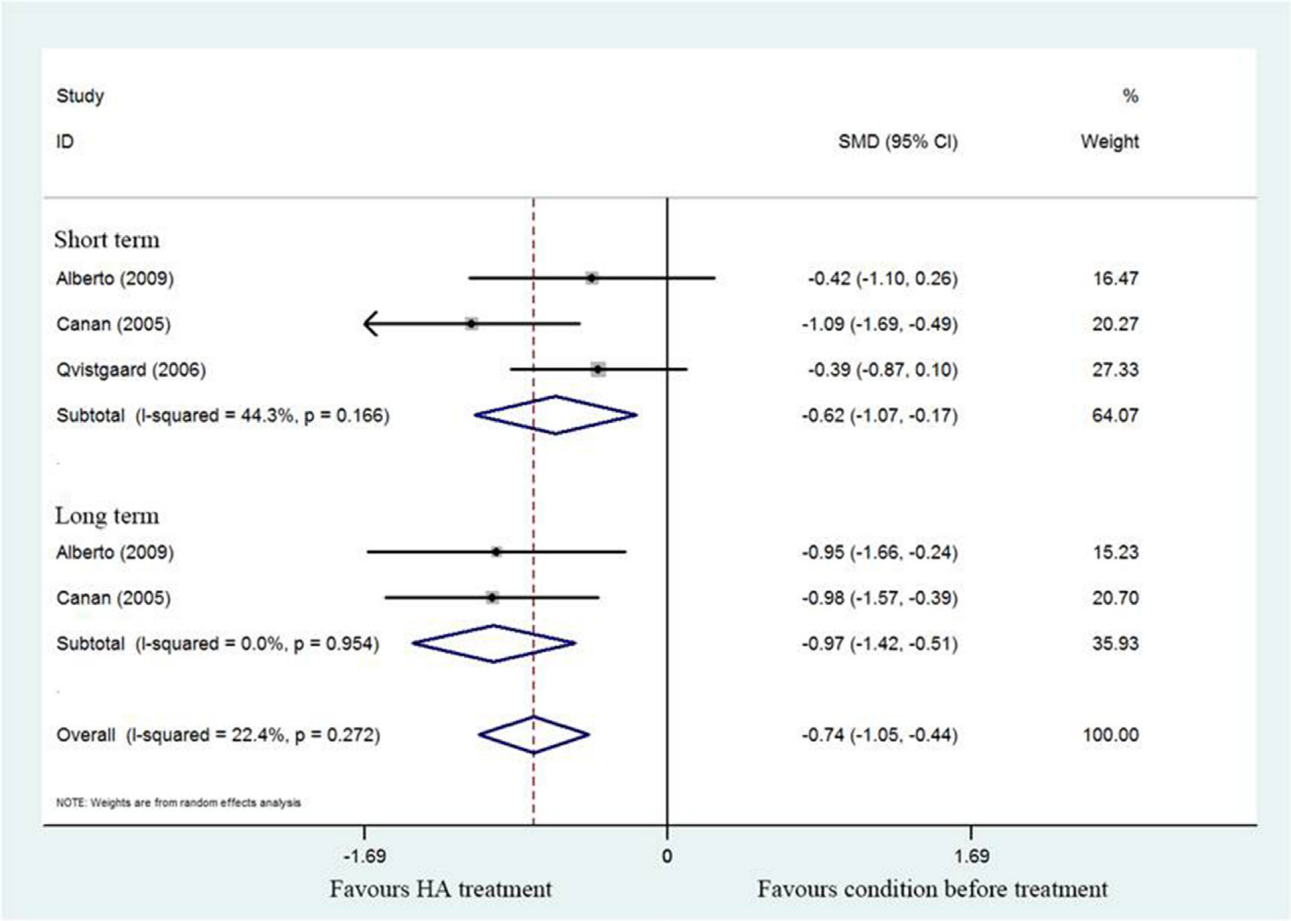
Forest plot showing different of standardized mean difference for Lequesne index between pretreatment and post-treatment

A total of three studies [17, 18, 20] revealed Lequesne’s index compared HA treatment with prior treatment. Notably, these 3 studies reported a significant difference in pooled Lequesne index (SMD = –0.74; 95%CI, –1.42 to –0.51; *P* < 0.05) with heterogeneity (I^2^ = 22.4%). As demonstrated in Figure 3, subgroup analysis of short- (SMD = –0.62; 95%CI, –1.07 to –0.17; *P* < 0.05) with significant heterogeneity (I^2^ = 44.3%) and long- term (SMD = –0.97; 95%CI, –1.05 to –0.44; *P* < 0.05) with no heterogeneity (I^2^ = 0%) of HA treatment also found a significant difference between post-treatment and pretreatment.

#### WOMAC function scores

**Figure 4:**
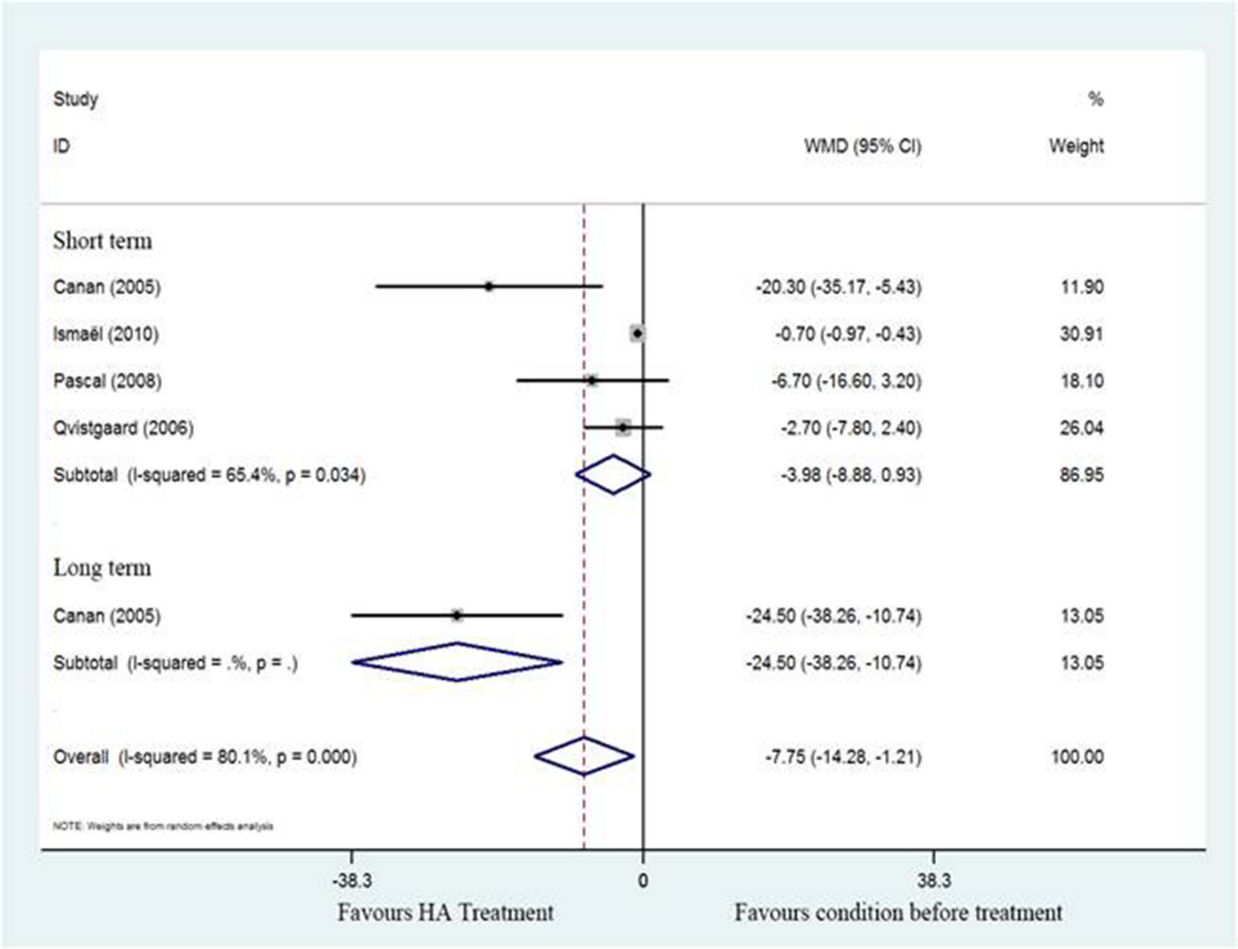
Forest plot showing different of standardized mean difference of WOMAC between pretreatment and post-treatment

The SMD for WOMAC scores in individual studies [17–19, 21] are detailed in Figure 4 (SMD = –7.75; 95%CI, –14.28 to –1.21; *P* < 0.05) with significant heterogeneity (I^2^ = 80.1%). However, subgroup analysis of WOMAC scores in short-term follow-up showed no significant different between post treatment and pretreatment using HA (SMD = –3.98; 95%CI, -8.88 to 0.93; *P* > 0.05) with significant heterogeneity (I^2^ = 65.4%). These findings suggest there was statistically significant decrease in WOMAC score associated with HA in long-term follow-up.

### Intra-articular injection of HA efficacy vs. control

#### Pain scores

**Figure 5:**
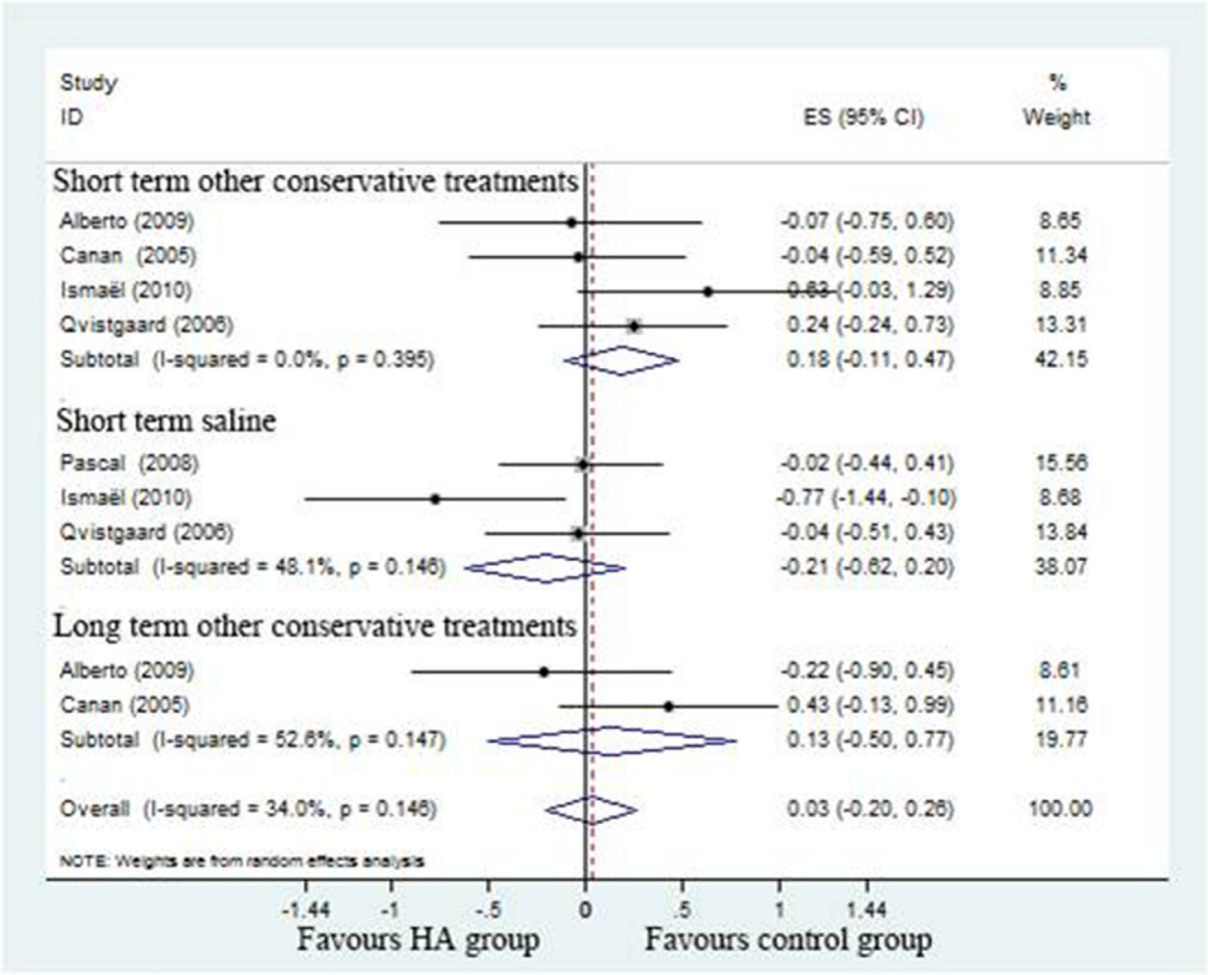
Forest plot showing different of effect size for pain between two groups

Five studies [17–21] compared HA with different controls, the pooled effect size of pain scores was 0.03 (95%CI; –0.20 to 0.26; *P* < 0.05) with moderate heterogeneity (I^2^ = 34%). Compared to positive control, the effect size of pain scores suggest no clearly different which was 0.18 (95%CI;–0.11 to 0.47) with no heterogeneity (I^2^ = 0%) in short-term and 0.13 (–95%CI, –0.50 to 0.77) long-term with significant heterogeneity (I^2^ = 52.6%). Compared to saline control, the effect size of pain scores was –0.21 (–95%CI, –0.62 to 0.20; *P* > 0.05) with moderate heterogeneity (I^2^ = 48.2%) (Figure 5).

#### Lequesne’s index

**Figure 6:**
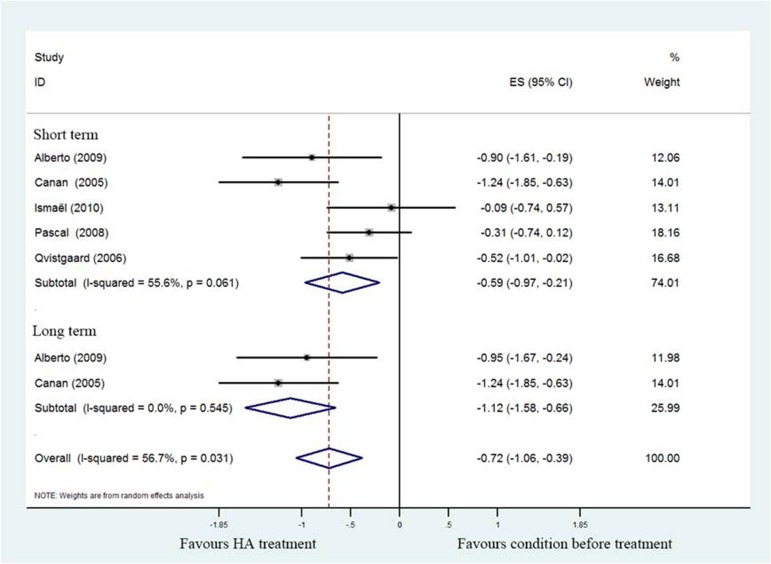
Forest plot showing different of standardized mean difference for Lequesne index between two groups

A total of three studies [17, 18, 20] revealed Lequesne index compared HA treatment with positive control. Notably, these three studies reported no significant difference in pooled Lequesne index (SMD = –0.24; 95%CI, –0.50 to 0.02; *P* > 0.05) with moderate heterogeneity (I^2^ = 20.0%). As demonstrated in Figure 6, subgroup analysis of short- term (SMD = –0.07; 95%CI, –0.40 to 0.26; *P* > 0.05) with no heterogeneity (I^2^ = 0%) and long- term (SMD = –0.29; 95%CI, –1.03 to 0.44; *P* > 0.05) with significant heterogeneity (I^2^ = 63.6%) These findings suggest there was no significant decrease in Lequesne index associated with hyaluronic acid therapy when compared with additional therapy.

#### WOMAC function scores

**Figure 7:**
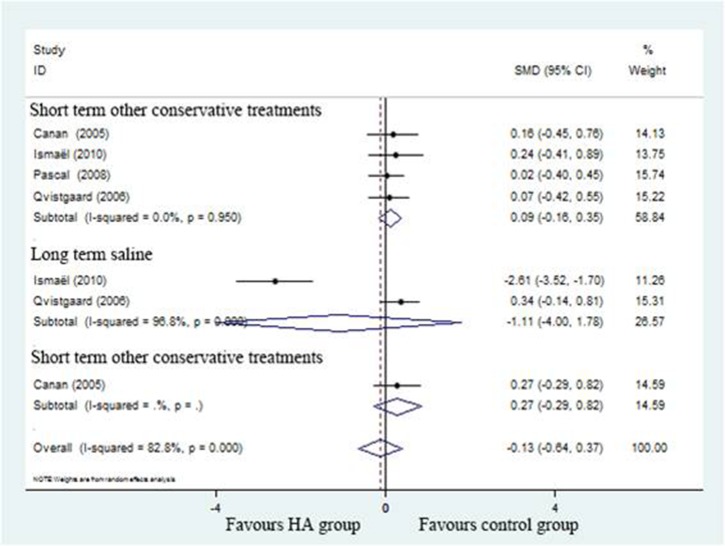
Forest plot showing different of standardized mean difference of WOMAC between two groups

The SMD for WOMAC scores in individual studies are detailed in Figure 7 (SMD = –0.13; 95%CI, 0.64 to 0.37; *P* > 0.05) with significant heterogeneity (I^2^ = 82.8%). Subgroup analysis of WOMAC scores compared to positive control (SMD = 0.09; 95%CI, –0.16 to 0.35; *P* > 0.05) with no significant heterogeneity (I^2^ = 0%) and saline control (SMD=-1.11; 95%CI, –4.00 to 1.78; *P* > 0.05) with significant heterogeneity (I^2^ = 96.8%) revealed there was no significant decrease in WOMAC score associated with HA group.

#### Adverse events

**Figure 8:**
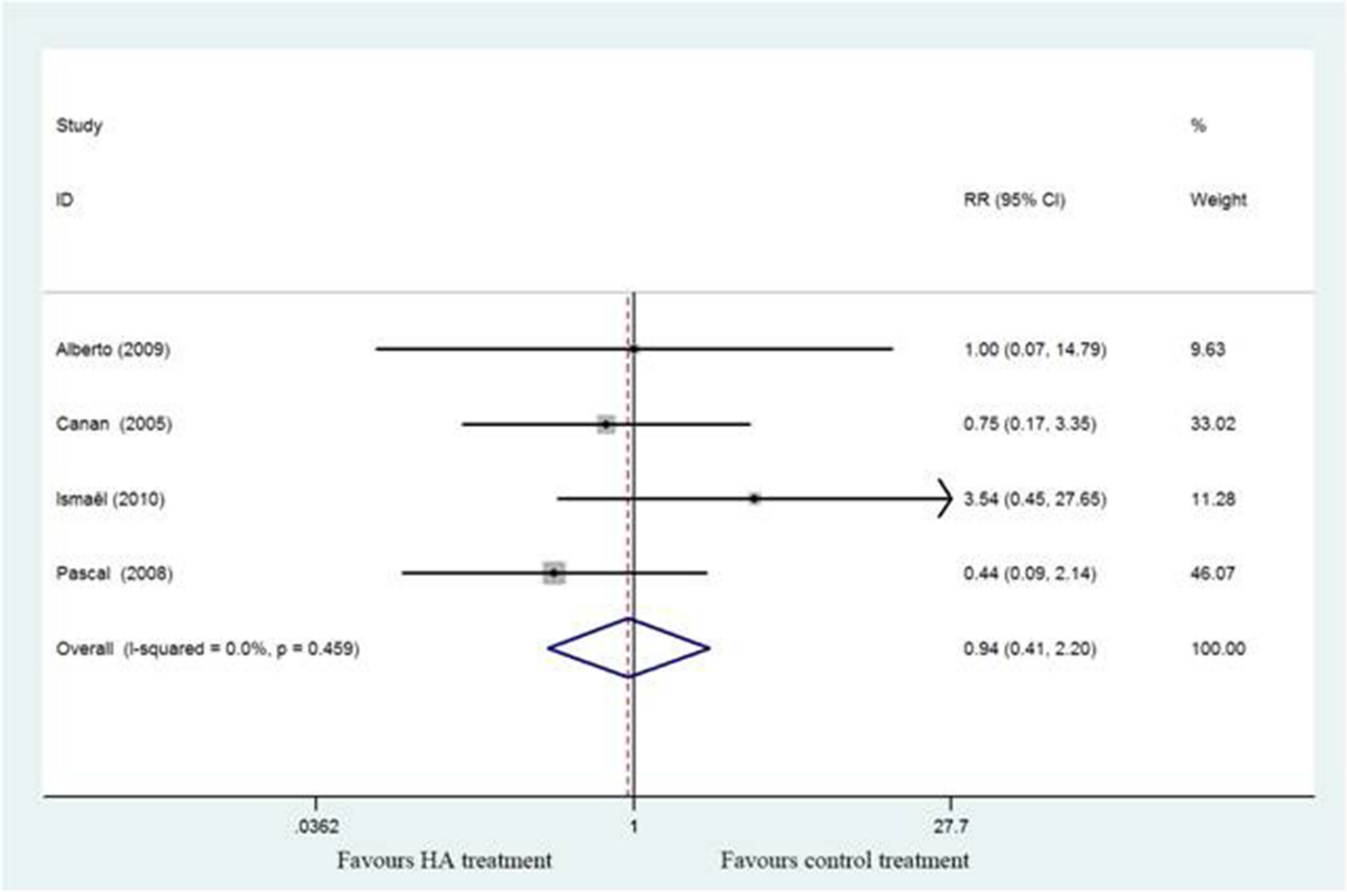
Forest plot showing different of risk ratio of adverse events between two groups

Five studies [17–21] describe the adverse events including transient post injection pain, superficial infection and hematoma and demonstrated there were no significant differences between HA and control group (RR,0.94; 95%CI, 0.41 to 2.20; *P* > 0.05) with no heterogeneity (I^2^ = 0%)(Figure 8).

## DISCUSSION

In this meta-analysis included six RCTs [17–22] aimed to investigate the effectiveness of intra-articular HA for hip OA treatment, and make any definitive conclusions about improvement scores of pain reduction, functional recovery and adverse events comparing baseline and control groups. The findings of our studies suggest a significant pain reduction based on pooled ES as well as a significant functional recovery improve based on pooled SMD of WOMAC function score and Lequesne’s index of improvement scores from baseline. However, the corresponding points estimate no statistical significance regarding the ES or SMD calculated from the difference between intra-articular HA and controlled therapies. We also indicate that there is no high risks of inducing adverse events by intra-articular HA for hip OA treatment.

The methodological quality assessment identified some limitations to the current evidence bases. The six RCTs satisfied with the defined eligibility criteria and the size of the comparative groups was small. A total of two studies[17, 18] reported the specific methods of randomization without referring random sequence generation and allocation concealment, allowing selection bias and two studies[17, 18] were lack of information for blinding of assessors allowed further measurement and expectation bias and the potential for type II statistical errors in measurements of these clinical outcomes. One study [20] reported a weakness rate of follow-up over 20% considered to have a incomplete outcome data. None studies was considered to have performed an intent-to-treat analysis. Heterogeneity may have been caused at the high risk of all types of biases because of variations in patient characteristics, different therapeutic strategies, and different strategies for measuring outcomes. Although we performed subgroup analyses stratified by follow-up time that cannot be completely resolved heterogeneity. None of the trials reported independent funding from any governmental or not-for-profit organization. Accordingly, this review of meta-analysis should be considered as conviction.

In this study, the effect size estimated by improvement scores from baseline in both short- and long- term follow-up indicated that intra-articular HA administration is an effective therapeutic approach for hip OA compared with the condition before treatment. Our results were accord with the general impression of joint injections of HA as a remedy in acute flares of activity in both rheumatoid arthritis and OA [23, 24]. In particular, the use of ultrasound-guidance in all included studies revealed facilitates an improved and accurate delivery of the injection, which is very important in the long-term management of hip OA [25]. The demonstration supports the hypothesis that suppression of inflammation responsible could reversible burden of pain and function, which has been considered as the promising strategies for preventing progression in osteoarthritis [19, 26]. Then, we speculated that dilution of intra-articular inflammatory mediator by fluid supplement may be partially responsible, thus contributing to treatment outcomes.

However, HA treatment seems to be not superior to placebo or conventional analgesic or pharmacological treatment against hip OA by measuring pain reduction. On the contrary, several studies on osteoarthritis of the hip indicated that the intra-articular use of HA products may be a relevant option in the management of patients suffering from hip OA with persistent pain, who do not respond to conventional analgesic or pharmacological treatment alternatives [27–29]. demonstrated that hyaluronic acid is more effective in the long-term, but corticosteroids are more effective than hyaluronic acid in the short-term [13]. From disputed results we speculate that reasons may as follow: Firstly, most studies demonstrated the benefit of HA in hip osteoarthritis were all open-label or included early osteoarthritis which had a lower credibility and higher risk of bias. Secondly, Qvistgaard et al suggest that HA did not have a current role to play in moderate to severe hip osteoarthritis and a similar tendency towards a relatively large effect of HA on patients with Lowish Kellgren grading on hip radiograms[18]. This is also probably a sample size problem due to the smaller number of patients included in these studies brings the one-sidedness, non-reprehensive outcomes.

The magnitude of response from normal saline (NS) from our meta-analysis is consistent with published data on the placebo effect in hip osteoarthritis, pain in hip OA could be relieved by injection of large quantities of NS and the short duration of response may be due to participants expecting their symptoms to worsen as they get closer to THR [30]. Another explanations is that the procedure of intra-articular injection of NS would favorably alter the abnormal joint environment benefits by diluting inflammatory cytokines and cartilage solution [31]. However, if saline may have an true influence on the attenuated symptom, we may choose the wrong negative control, creating a smaller difference than practical situation.

We also concluded that intra-articular treatment with HA has a significant therapeutic efficacy in patients with hip OA on functional recovery compared with baseline measured by Lequesne index and WOMAC function scores and predicted that there was a long-lived effect. Cochrane et also reported that HA is an effective treatment for knee OA, with beneficial effects on function, and patient global assessment [23]. An improvement in the Lequesne index and WOMAC function scores might also lead to improvements in common activities of the patient, such as work and self-care. These results encourage pharmacoeconomic studies to establish precisely the cost-effectiveness of intra-articular treatment in the management of hip OA. In the meantime, HA treatment seems to be not superior to placebo or conventional analgesic or pharmacological treatment against hip OA from our results. We considered that pooling of different treatment strategies and different grades of osteoarthritis of the hip is not ideal for a comparing study. Another limit is the fact that we only compared the efficacy of intra-articular administered hyaluronic acid at six months and not longer, which may not distinguish different efficacy from other pharmaceuticals [23].

Another notable finding from this meta-analysis is that intra-articular HA products are not associated with increased safety risks. This is in sharp contrast to Rutjes et al who speculated that HA increased the risk of serious adverse events and adverse events-related subject withdrawals [32]. Reichenbach et al. had also indicated that HA products are associated with a greater frequency of local acute inflammatory flares [33]. Therefore, this treatment should use to painful hip OA. And the therapy still needs a large long-term trial to clarify the benefit-risk ratio with clinically relevant.

Limitations of this meta-analysis included, Insufficient statistical power to distinguish with the clinical important differences; and the significant heterogeneity existed across studies resulted from differences in study sample sizes, pain assessment, regimens, preparations of HA, and reference treatments. Consequently, we chose to extract scores from pain scales and standardized them using the effect size to allow for a quantitative analysis. However, relative risks used in this meta-analysis minimize the impact of discrepancy of definitions of local adverse events. Strengths of this meta-analysis are Not only that we conducted a rigorous literature search of RCTs with high quality included, but also set effectual validity of estimates and conclusions drawn from the meta-analysis. Comparing previous mete-analysis, we have additional compared the function recovery scores which more sufficient to describe the intra-articular HA treatment in hip OA.

## CONCLUSIONS

This meta-analysis results suggest that intra-articular HA in hip OA can significant reduce pain and improve functional recovery when compared with baseline. However, there seems no significant different between HA and saline or other treatments. And the currently available evidence indicated that intra-articular HA in hip OA would not increased risk of adverse events. Therefore, for future studies, it is relevant to determine the risks and benefits of HA for treating hip OA by large, well designed RCTs comparing different drugs of intra-articular administered.
